# News and Coming Events

**Published:** 1907-05-18

**Authors:** 


### NEWS AND COMING EVENTS.

The King has sent a present of linen to the Chelsea Hospital for Women.

The King's Hospital Fund Bill was read a second time in the House of Commons on May 13.

At the thirty-ninth annual banquet of the French Hospital in London, it was announced that the amount received in response to the appeal for funds towards the enlargement scheme totalled £4,490.

Professors Von Leyden and Erb, of the universities of Berlin and Heidelberg respectively, have been elevated to the rank of Privy Councillors of the First Class, with the title of Excellency.

Dr. Alfred Howell has been appointed honorary assistant physician to the Cardiff Infirmary, in the place of Dr. Arthur Taylor, who has been appointed honorary physician to the institution.

The Norrisian Professor of Divinity at Cambridge (Mr. F. C. Burkett) has received the honorary degree of D.D. from the University of Edinburgh. Although he is a member of the Cambridge Theological Faculty, his own university has found it impossible to confer a similar honour upon him—because he happens to be a layman.

Hebburn owes a good deal to the help given by the late Mr. and Mrs. Arthur Coote, who interested themselves in almost every local agency for the betterment of the people, and particularly in the local infirmary. Lately, two portraits of these two benefactors of the Hebburn Hall Accident Infirmary, presented by the family, have been unveiled.

With reference to the retirement of Dr. Frederick Taylor from the post of senior physician to Guy's Hospital, we are requested to state that he was appointed assistant physician in 1873, and that his total service to the hospital as assistant physician and physician has been thirty-four years. If his student days are included Dr. Taylor's connection with Guy's dates back upwards of forty years.

Middlesex Hospital has many influential friends, who assembled in large numbers to support the Duke of Connaught at the festival dinner on May 10. For 150 years Middlesex Hospital has done yeoman service to the suffering and the sick. It contains 341 beds, and treats 40,000 out-patients annually. One of the most useful sides of the work done at Middlesex Hospital is the cancer charity. Here 159 in-patients and 47 out-patients are under treatment. It would be difficult to over-estimate the services which this department has rendered to the community since it was first opened in 1792. The Duke of Connaught made a most eloquent appeal, which resulted in subscriptions amounting to £4,500.

The West End Hospital for Nervous Diseases, 73 Welbeck Street, W., is appealing for funds in aid of its extension scheme. On an average 64,000 out-patients' attendances are registered annually, and there are necessarily a great number of adult patients who require indoor treatment; 25 adult beds have been provided at a cost of about £2,000. As the space in the hospital is so limited, and there was no means of acquiring any of the adjoining property, the accommodation for these beds has been found as near the hospital as possible. The committee has purchased a site for £2,500. For some years past the nursing staff have had to be housed some distance from the hospital, going to and fro to their work. With the change proposed, accommodation can be found for the nurses also.

The Out-Patient Visitors' Report of Guy's Hospital states that of 41,118 new out-patients interviewed in 1906, and from whom particulars of their circumstances in life were obtained, only 124 persons were found ineligible to receive the benefits of the charity.

H.R.H. Princess Louise, Duchess of Argyle, has been especially zealous in the cause of the hospitals during the last few weeks. On May 14 she opened the new out-patients' hall in connection with the London Temperance Hospital. The Chairman of the Hospital, Sir Vezey Strong, presented Princess Louise with an address, which was also signed by the treasurer and hon. secretary. 25,216 out-patients, or a daily average of 218, were treated at the hospital during 1906. £3,000 is still required to discharge the whole of the liability incurred by the erection of the new buildings.

The Festival Dinner of the Royal Waterloo Hospital for Children and Women at the Savoy Hotel on May 14 was a very pleasant function. The Duke of Argyll and H.S.H. Prince Alexander of Teck showed great interest in the institution, which has recently been rebuilt and has only a third of the ward accommodation occupied at present owing to want of funds. The new wards are airy, light, and pleasant, but the out-patient department is badly planned, and, in view of the approaching summer weather, should be rearranged without delay. The Secretary, Captain Houston, has worked very hard for the dinner, which produced £1,300.

An electric motor ambulance service for the City was inaugurated on Monday, May 13, by Alderman Sir A. Newton, Chairman of the Police Committee. A temporary ambulance station is near St. Bartholomew's Hospital and King Edward Street, Newgate Street. It is estimated that two thousand accidents happen each year in the City streets, and fifty-two call-boxes have been erected at the most convenient centres, to enable the police on duty at the spot to instantly communicate with headquarters and with the motor ambulance station. Practically the whole of the City Police force are first-aid men. The new ambulance is of eight-horse power, and can carry two patients in a recumbent position and three others in a sitting posture. If the motor ambulance proves successful, two others will be added in due course.

The new wing and extension of the Glasgow Samaritan Hospital for Women have just been opened. The new wing contains four large wards with accommodation for ten beds each, and four smaller wards for three and two beds each, two large convalescent rooms, two well-equipped operating rooms with surgeons' rooms attached, two ward sculleries, and ample lavatory provision. The extended hospital now contains accommodation for eighty-six beds, and is the largest women's hospital in the United Kingdom. The administrative block between the old and new wings has been entirely remodelled. A new doorway and large entrance hall has been provided. A new office occupies the old board room. The new board room adjoins the lecture room and is separated from it by folding partitions, and when these are thrown open a hall is formed with accommodation for 150 persons. Provision will also be made for the meetings of the Ladies' Auxiliary Association and the work of the Dorcas Society. The cost of building and furnishing the new wing and operating rooms, together with the other additions and alterations, has amounted to over £12,000, while the total cost of the whole hospital buildings, including nurses' home and dispensary, with their equipment and furnishings, has been about £40,000.

